# Chronic Dislocations of the Shoulder

**Published:** 1875-12

**Authors:** W. W. Miner


ART. II.—*Chronic Dislocations of the Shoulder. With Observations upon the Manipulation Method in Reduction of the Shoulder Dislocation.* By W. W. MINER, M. D.

The first and greater portion of this article was read as an essay before the Buffalo Medical Association on November second, 1875. In offering it for publication, it is my pleasure to acknowledge my indebtedness to my uncle especially, and to my father, both mentioned herein, for many important opportunities of observation of the reduction of old dislocations, as well as for many ideas I have advanced upon this subject.

The histories of two cases are here presented, of chronic dislocation of the humerus, in whose restoration I assisted, which have not been reported and which present some points of interest differing from those of other cases with which I have had to do. Following these, are remarks upon such cases in general: I add in conclusion, some observations I have gained from dissecting room studies respecting the reduction of the shoulder dislocation by the manipulation method.* Little is said by authors of surgical works on the subject of chronic dislocation of the shoulder, and personal experience in this respect, is of more than usual interest.

CASE I.—J. S——, an Irish laborer, aged about forty-five, came to Dr. J. F. Miner, from Chautauqua county, with a dislocation of his right shoulder. He was suffering considerably from pain and

---

* Report of a Case of Reduction of a Shoulder Dislocation of seven months standing, by Prof. J. F. Miner, in which I assisted, also of a Congenital one of eleven months, is given in an excellent article by Dr. E. N. Brush, in the BUFFALO MEDICAL JOURNAL for October, 1875, and reference is therein made to other like cases by Dr. Miner, reported in that publication.

numbness. It was four weeks since the injury had occurred. Ineffectual effort at restoration had been made a short time before his coming here. He took quarters at the Sisters of Charity Hospital, and with others came before an invited assembly of Alumni and friends of the Medical College, for operation, on February twenty-third, 1875. He had entire immobility of the arm, a flattened shoulder and constant harassing pain through the length of the limb. The diagnosis of sub-glenoid dislocation was made with unanimous consent.

Anesthesia with ether being thoroughly procured, Prof. J. F. Miner selected assistants from the company and proceeded to accomplish reduction. The patient was lying flat upon an operating table, a sheet was made fast at the elbow and entrusted to several for effort at extension in the axis of the arm. Another sheet was made to pass under the axilla, by which counter-extension and traction of the head of the humerus upwards and outwards, was to be sought. The operator grasped the forearm and elbow in his hands, placed his heel in the axilla, and while extension and counter-entension were being energetically made, he carefully made rotation of the arm. The extension was varied from directly downwards in the axis of the body, to downwards and forwards, the counter-extension varying to correspond. A process of circumduction, limited in area, was carefully conducted in combination with a degree of rotation. Careful pressure downwards upon the acromion was also made. A number of professional gentlemen were vigorously occupied in making the extending and counter-extending force A giving way of adhesions was repeatedly felt and the bone arose to its place without marked suddenness of movement.

After the sheets for traction were removed, the shoulder beneath the acromion, was found to have gained rotundity, though not quite to the degree which was thought naturally present. It was, however, such in degree as had been before noticed to be present immediately succeeding restoration in such cases. It was the opinion of the profession present that reduction had been made. On motion of the arm, which was free and unobstructed, there was now present a grating sensation, as of the rubbing together of roughened joint surfaces, which was very manifest to those who made after examination of the limb. It was considered that inflammatory deposits of more than usual abundance had occurred and hindered perfect apposition of the joint surfaces, and that this, with an atrophied condition of the deltoid, explained also whatever defect of contour there was in the shoulder. In order to have incontrovertible evidence that the dislocation was reduced, I tried what is known as Dugas' test, brought the hand to the opposite shoulder, and even to the head. There was no ground for the question of fracture, I was entirely satisfied that the shoulder was as near its normal condition as the fibrinous deposits upon the head of the humerus and the glenoid cavity of the scapula, would allow.

His elbow was bandaged to his breast to avoid all danger of redislocation and motion thus prevented for a week afterward. The bandages were then removed. He was entirely free from the pain and numbness he had had. Though the deformity of his shoulder was such on entrance to the Hospital that his condition was diagnosed by the hand, before his clothes were removed, there was a scarcely noticeable want of fullness now. The man was one who thought very little about his arm in comparison with being hurt, and therefore the motion I made was restricted somewhat, still it was sufficient to show me that there was a satisfactory condition in this respect. There was present likewise the same rubbing, grating sensation, as distinctly felt on moving the arm, as before. It gave the patient no pain to produce this rubbing movement, and he was altogether free from pain when let alone. I advised and urged him to make passive motion of the shoulder himself by grasping something as high as his shoulder and then bearing down gradually with his weight.

At the end of another week, fearing less through my inability to secure satisfactory motion and his indisposition to this exertion the function of the joint should become permanently impaired, I advocated anesthesia and proper motion of the joint, but this was not brought about. He remained a week or two more, then went home. I trusted that return to his labor would secure him tolerable use of the joint. On May first, two months after the operation, I found him returned to the hospital, Dr. Cronyn being in charge. The same roughened character of joint surfaces was present, and motion was restricted to quite a small compass. This condition persists at present I have no reason to doubt.

The result here I do not think a surprising one. I had not seen such result occur in other cases. I did not expect it would develop here to the extent it has. There is generally, I believe, a slight impairment of the motion of the shoulder which persists, but which is not of great inconvenience in any of the ordinary uses required of the arm. A case which attracted interest and was brought to the attention of quite a number of physicians of this city, last winter, was one that presented similar characteristics on motion of the shoulder joint to that so plainly noticeable here after reduction. There was markedly manifest on motion of the arm, a roughened, grating sensation, as if irregular surfaces of bone were being rubbed upon each other. It caused question in some as to whether there was not dislocation, or fracture of the head of the humerus; some expressed their opinion that one of these conditions was present. There had been no injury that was supposed at the time to have produced dislocation, there was mobility and absence of nearly all ill effects of dislocation. The elder and experienced of the observers recognized a roughness of the joint surfaces, which though very marked in degree, was such as they had repeatedly witnessed as the result of simple inflammatory action. The condition of roughened joint surfaces in the foregoing case, I take it gave evidence that the inflammatory action accompanying the dislocation was of unusual degree of severity and ought to have indicated particular caution in the after treatment by passive motion.

Case II.—In the fall of 1870, I assisted Dr. D. W. Miner, of Ware, Mass., my father, in reducing a neglected dislocation of the humerus of six week's standing. The patient was Mrs. J. B——, of about thirty-seven years of age, who had injured herself by falling from a wall. Three different physicians had been in attendance and had consulted together concerning the case, had treated it as fracture of the neck of the humerus, but not having satisfactorily disposed of it, they requested Dr. Miner to visit her.

On arriving at her home in North Dana, some fourteen miles distant, the patient was found to be a fleshy person, who had had since her fall, complete disability in her left arm, She had not had much pain in it, but it was numb, almost immovable, and was kept constantly fixed by her side, with the elbow thrown a little out from the body. There was a depression readily felt beneath the acromion but was not marked in the contour of the shoulder, on account of her fleshy condition.

In reducing the dislocation an apparatus called Jarvis' Adjuster was used. This is a simple contrivance for producing any desired amount of extending force by aid of a rack and pinion movement. One extremity was attached to the elbow on its inner side with bands of cloth, the other having a leathern stirrup with suitable padding, was applied to the axilla. The position of the arm was not thereby changed at all from that it had before maintained by the side and nearly in the axis of the body. After anesthesia was obtained and satisfactory extension produced, the forearm was grasped by the operator at the wrist and elbow, and thus careful motion of the shoulder made. Bands of adhesion gave audible snaps as the extension was increased, and the movements of circumduction and rotation renewed. The bone rather suddenly slid up to its natural position. After removal of the instrument for extension, it was found that there was a noticeable improvement in the appearance of the shoulder. No great importance was attached to the after treatment by passive motion. Three months afterwards the result was found to be entirely satisfactory.

The manner in which the force of extension was applied in the above case, appeared to me unobjectionable, and has some features in which it may at times be more advantageous than others. The force is applied in a way very easy for the operator and can readily be increased or regulated to any desired extent. As little injury is done to the soft parts as by any other methods, it makes no show as of extreme measures. Jarvis' apparatus is not often used because of its limited adaptibility and because professional assistance is usually available. In case the operator has no experienced assistants he could in this way accomplish what he desires without them. I have certainly never seen such an operation performed with greater ease than it was in this instance.

General Remarks.—The ease with which reduction of old dislocations is accomplished, I have found varies considerably in different cases. The greatest difficulty was experienced in the case of a blacksmith, sixty years of age, whose shoulder had been out of place for ten weeks. It was only after resolute and fatiguing effort by five or more able men, that the desired result was obtained. The patient had soreness of the arm and felt the after effects of the operation somewhat for a few days, but gained very satisfactory use of the shoulder without any particular care in after treatment. Perhaps the effort required in the case of the laborer whose history I present here; was next in importance. An abundance of help being at hand, it was made use of. In the case of the farmer reported by Dr. Brush, of seven months' standing, which Dr. J. F. Miner reduced a short time since, though the effort was quite energetically made, its amount and the time occupied, was much less than in those mentioned. I accounted for this in the fact that this patient, though owner of a farm, was not as much of a worker as were the others, had not the same brawn of muscle or same degree of ligamentous development. Quite various considerations are necessary in determining the irreducibility of an ancient dislocation.

Complete anæsthesia is as necessary in these chronic cases as in recent ones. In the case of La Montaine, the blacksmith, whose case is reported in the Buffalo Medical and Surgical Journal of December, 1874, after traction had been made in the various direction of downwards, forwards, inwards, very free circumduction was made, while the extension force was held up, with a view to rupturing thoroughly any bands of adhesion that might obstruct the return of the bone. Afterwards when traction was resumed, the patient being on the floor, was at my suggestion turned from his back over upon his sound side and the operator brought the arm backwards, behind the line of the body and made rotation, extension being then downwards, backwards and inwards. This position is I think most favorable for reduction in recent cases, and in this case the return was effected, if not behind, quite near to the line of the axis of the body.

I have seen very strong effort made in this method of reduction, have had at such times, fears of the accidents that have repeatedly happened in these operations, but I have never myself seen any important ill effect whatever occurring or resulting therefrom.

I believe the method in which the traction was applied in the cases herewith cited is less likely to cause acccidents than other methods suggested. For examples I should consider the method advanced by Dr. Kirby, of Dublin, and numerous others open to great objection. By it, traction with pulleys is made outwards, more or less in the line of the axillary vessels and nerve, and if these are bound down by inflammatory adhesions, they would thus be in great danger of violence.

The use of leverage in the long bones, is of limited practibility. Although it is a proper and effectual method for restoration in many cases, still it is often attended with unfortunate results, and should always be employed with discretion. The great mechanical force exerted thereby upon the short end of the bony lever, transversely to the line of its shaft, suffices frequently to occasion fracture of an articular process, at its extremity, (e. g. olecranon,) of the neck, or of the shaft of the bone. Though such an occurrence is sometimes of not great importance in its after results, it may cause a condition worse than that for whose relief the effort was instituted. I have found that direct traction and countertraction in the line of the dislocated bone, combined also if desirable with moderate presure or traction with a third band, obliquely thereto, can be made with all the energy that can be judiciously applied towards reduction of joint surfaces; that such effort can be made thereby with the minimum of danger to the bones, and by attention thereto, to the accompanying vessels and nerves.

If fracture of the head of a bone is complicated with unreducible dislocation of the same, I have reason to believe that removal of the head is preferable to other procedures which have been made or suggested.

The remarkably excellent results I have witnessed of late from passive motion, not alone in inflammatory disturbance of the ligamentous structures of a joint, but as well of the synovial surfaces also, has very greatly increased my appreciation of its value and importance.

Last spring while pursuing anatomical studies, I took occasion to observe the shoulder joint particularly with reference to its dislocation. I queried whether it bore any similarity to the hip joint and whether manipulation might not be an efficient method in the reduction of dislocation here as it is in the hip joint. The axillary dislocation is readily produced, with the fingers I found in the cadaver, and especially if the tendon of the deltoid has been severed. The capsular ligament which surrounds the articulation, holds the humerus only quite loosely. The thumb will easily push the head of the bone downwards and forwards into the axilla, when the loose character of the ligaments of the joint is very evident. The only part of the ligaments of this articulation which are of great strength, is that ligamentous band which passes from beneath the coracoid process downwards and outwards to be attached to the groove which marks the anatomical neck of the humerus and at a point near and on either side of the location of the tendon of the biceps muscle. When the humerus is in place this ligament runs obliquely downwards and outwards and is tense; it then prevents extreme eversion of the head. When the humerus is dislocated, the two points of attachment of this ligament, are approximated nearer each other, and this strong band becomes lax and has no more power than the capsular ligament does ordinarily.

By rotating the arm inwards so as to bring the anterior face of the head of the humerus into bearing upon the articular head of the scapula, you secure a position of the humerus favorable to the occurrence of the sliding movement of restoration. Now by carrying the elbow back, you may render tense this strong ligament, the coraco-humeral, and secure thus great power towards reduction. In fact reduction will occur or rupture of the ligament, and this in all ordinary cases would not happen. I believe this is the rationale of reduction by manipulation in the shoulder. The subject has been advocated by Henry H. Smith in this country, and is mentioned with favor by Gross in the last edition of his work.

Since reading the above on November second, before the Buffalo Medical Association, my attention has been called to an article in the *Monthly Abstract* for November, which I take liberty to present herewith :

*On the Analogies of Dislocation of the Shoulder and Hip-joints, and the Methods of Reducing them.*

Dr. Kocher contributes, in Volkmann's *Sammlung Klinischer Vortrage,* an article in which he bases the methods of reduction of dislocation of the shoulder and hip on the anatomical structure of the joints. He remarks that these joints possess several points of analogy, especially in their ligamentous apparatus. The Y-ligament of the hip-joint, which proceeds from the anterior inferior spine to the linea intertrochanterica, and is connected with the zona orbicularis, has its analogue in the coraco-humeral ligament, which, arising from the coracoid process, divides into two branches, one of which is inserted into the greater tuberosity, the other into the lesser tuberosity of the humerus. From both these branches fibres proceed to the capsule, and perform the same functions as the orbicular ligament. These anatomical analogies indicate a similar mode of reduction. The dislocations of the shoulder and hip are essentially either forwards or backwards.

1. In dislocations upwards and forwards (subcoracoid and ilio-pubic), the special movement for reduction is flexion, which in the case of the shoulder must be preceded by strong rotation outwards, while in the case of the hip this has already been done. After flexion, rotation inwards and extension follow. In the hip, extension follows immediately on flexion.

2. Dislocations downwards and forwards (axillary and obturator) require rotation outwards, which must be preceded by flexion and traction.

3. Dislocation downwards and backwards (infraspinous and sciatic) require rotation inwards, flexion, traction, and finally rotation outwards.

4. Dislocations upwards and backwards (sub-acromial and iliac) require flexion or the utilization of that already existing, traction and rotation inwards.

The methods under consideration are essentially those of elevation and rotation. Elevation serves either for relaxation of the stretched portions of the capsule (the ilio-femoral ligament by flexion, the coraco-humeral ligament by flexion and abduction) or for stretching them so as to form a firm point for leverage.

Irregular and old dislocations require somewhat modified procedures.

The use of chloroform in the reduction of dislocations should be limited, according to Kocher, as muscular contraction may often be useful.—*London Med. Record,* Sept. 15, 1875.

You will notice the agreement of Dr. Kocher's anatomical observations with those I had recorded in my note-book and presented before the Association. The practical deductions of my fellow observer are very finely drawn and the analogies quite attractive. In my opinion, however, they are not practical in such degree as to be useful.

In the method advocated and practiced by Prof. Henry H. Smith, elevating the arm is the first movement directed. I think this is a mistake. Not only is it impracticable to raise the elbow, in dislocation into the axilla, on account of the pain it causes the patient, but on the cadaver it may be noticed that this movement of the elbow upwards, makes tense the axillary vessels and nerves to an unsafe degree if done as one would infer from the directions. In raising the humerus to a horizontal position, the dislocated head forms a promontory, jutting down below the usual line of course of the axillary vessels and nerve, and the movement up gives a leverage power of tension upon these, which is a source of danger and could hardly fail to cause unfortunate results if extreme elevation was made. In neglected dislocations where fibrinous adhesions limited the elasticity of these axillary cords, of course, the necessity for caution is much increased. Again, it is my opinion that this elevation recommended by Crampton and still later writers, is not necessary.

Lacour's method by which Prof. Gross says he "reduced in a few seconds in 1869, after failure of other means, an axillary dislocation of nearly three months standing," accords with that I found most efficient and advisable. However failure might occur in its use because the backward motion still beyond the line of the body, is not advocated. To describe the movement I would recommend in a way that cannot be mistaken or forgotten—I term it a movement of the dislocated extremity as if one wished to draw his handkerchief from his coat-tail pocket. This tells the whole procedure. In this you notice inversion of the humerus is produced with the object of securing a favorable surface, the anterior face of head of the humerus, for the sliding movement of restoration.

This object can be accomplished by outward rotation, that is, eversion of the humerus, but this is not so desirable or so easily obtained a position. In this backward movement the elbow may be carried as far behind as is required, and the hand even beyond the spine. In this way the coraco-humeral ligament is made tense and powerful force brought to bear towards restoration.

